# Inter-individual differences in serotonin and glutamate co-transmission reflect differentiation in context-induced conditioned 50-kHz USVs response after morphine withdrawal

**DOI:** 10.1007/s00429-018-1683-4

**Published:** 2018-05-17

**Authors:** Adam Hamed, Miron Bartosz Kursa

**Affiliations:** 1 Laboratory of Spatial Memory, Department of Cellular and Molecular Biology, Nencki Institute of Experimental Biology, Polish Academy of Sciences, 3 Pasteur Street, 02-093 Warsaw, Poland; 2 Interdisciplinary Centre for Mathematical and Computational Modelling, University of Warsaw, Pawinskiego 5A, 02-106 Warsaw, Poland

**Keywords:** Amygdala, Morphine, Reward, Context conditioning, USVs, Ultrasonic vocalization, Incubation of craving, Neurochemistry, Machine learning, Serotonin, Glutamate, GABA, Nucleus accumbens, 5-HT, Co-transmission, Inter-individual differences;, Context-induced, Glu/Gln ratio

## Abstract

A growing body of research provides compelling evidence that in rats 50-kHz USVs are a form of expression of positive emotions. Context-induced 50-kHz USVs emission is variable among rats, indicating individual differences in contextual response bound up with pharmacological reward. The aims of this study were to: extract the most important neurotransmitters related to context-induced conditioned 50-kHz USVs response; find biological basis of existing inter-individual differences in context-induced conditioned 50-kHz USVs response; create a model of all-to-all neurotransmitters correlations. The data collected here confirms that re-exposure to the context of morphine administration after the withdrawal period increases the level of 50-kHz USVs and this contextual response is associated with elevated serotonin concentrations in amygdala, hippocampus and mPFC and with increased Glu/Gln ratio in nucleus accumbens. The concentration of serotonin increases simultaneously in amygdala, nucleus accumbens and hippocampus. Moreover, 5-HT concentration in amygdala is bound up with glutamate level in this structure as well as in hippocampus. Furthermore, Glu/Gln ratio in nucleus accumbens has strong associations with Glu/Gln ratio simultaneously in VTA, amygdala, striatum and hippocampus. *All-to-all*-analysis indicate that concentration of glutamate in hippocampus is proportional to glutamate in VTA and GABA concentration in the hippocampus. We have also demonstrated that Glu/GABA ratio in VTA and amygdala was elevated after *post withdrawal* re-exposure to the pharmacological reward paired context. Presented analysis indicates a strong correlation between serotonergic and glutamatergic systems in context-induced conditioned response. The strength of this co-transmission correlates with the number of 50-kHz USVs emitted in response to morphine-paired context.

## Introduction

A modern behavioral tool that most effectively determines the emotional states of rats is the registration and analysis of ultrasonic vocalizations (USVs). It allows both identifying individual differences in processing information about the reward as well as reflecting, to a large extent, the level of individual motivation. It is well established that rats emit two district ultrasonic vocalizations patterns (USVs) related to separate emotional tinge: long 22-kHz alarm calls when processing negative emotions and 50-kHz when processing positive emotions (Panksepp and Burgdorf 2003; Brudzynski 2013a, b; Burgdorf et al. 2017). Representations of these sounds are also present in rats’ social interactions (Knutson et al. 1998; Brudzynski and Pniak 2002; Hamed et al. 2009, 2015). The mesolimbic reward system is highly involved in the production of 50-kHz ultrasonic vocalizations (USVs) in rats (Knutson et al. 1999; Burgdorf et al. 2001, 2007). Large number of studies indicate that the most potent pharmacological agent that induces 50-kHz USVs in rats is amphetamine (Burgdorf et al. 2001; Thompson et al. 2006; Wang et al. 2008; Ahrens et al. 2009; Wright et al. 2010; Brudzynski et al. 2011; Simola et al. 2012; Taracha et al. 2012). In a recent study we conducted non-parametric analysis of neurochemical effects showing that re-exposure to amphetamine induces neurochemical changes in several brain areas (Hamed et al. 2016). We demonstrated that increased concentration of noradrenaline in the nucleus accumbens strongly correlated with the number of 50-kHz USVs (Hamed et al. 2016). Another set of pharmacological experiments demonstrated that noradrenaline related mechanisms are highly involved in 50-kHz USVs emission (Wright et al. 2012a). Previous studies have established that intracerebral injection of glutamate may induce 50-kHz USVs, suggesting that glutamatergic system is also involved in production of these sounds (Fu and Brudzynski 1994; Wintink and Brudzynski 2001). Additionally, administration of the amphetamine with MK-801 (NMDA antagonist) suppressed emission of the 50-kHz USVs compared to amphetamine treated animals, suggesting that glutamatergic system may be highly engaged in 50-kHz USVs emission (Costa et al. 2015). Co-administration of those two substances suppressed likewise context-induced conditioned 50-kHz USVs when animals were re-exposed to the drug-paired chamber (Costa et al. 2015). Moreover, there are evidence showing that antagonism of 5-HT 2C or the κ-opioid receptors increases emission of 50-kHz USVs (Wöhr et al. 2015; Hamed et al. 2015). Furthermore, agonist of 5-HT2C receptor suppressed amphetamine induced sounds in this frequency band (Wöhr et al. 2015).

**Fig. 1:**
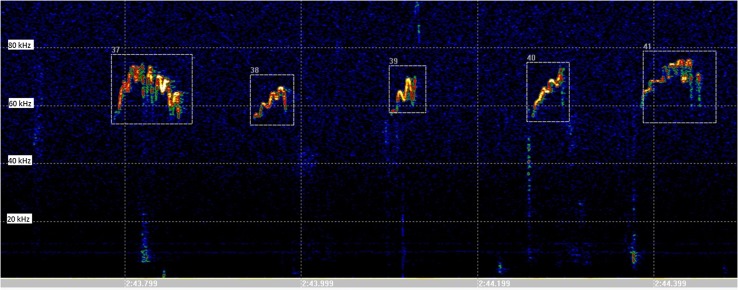
An example of 50-kHz ultrasonic vocalization episodes

A growing body of research provides compelling evidence that 50-kHz USVs (Fig. 1) are a form of expression of positive emotions. However, pharmacological studies indicate that processing of positive emotions is not always accompanied by the 50-kHz USVs in rats. In case of morphine treatment, higher doses of this addictive drug (10 mg/kg) decreased the sounds triggered by social interaction (Hamed et al. 2015). It has been also demonstrated that MDMA, which induces huge arousal (excitation) in humans, did not evoke an increase in total number of 50-kHz USVs (Sadananda et al. 2012; Simola et al. 2012).

In the canonical conviction, dopaminergic reward system, originating from ventral tegmental area (VTA) is crucial for reward processing (Schultz et al. 1997; Dayan and Balleine 2002; Cohen et al. 2012; Lammel et al. 2012). The latest reports have shown that another structure—dorsal raphe might be equally treated as a key structure in reward processing and reward expectation (Luo et al. 2015, 2016; Li et al. 2016; Matthews et al. 2016; Qi et al. 2014). In mice with dopamine deficiency SSRIs (selective serotonin reuptake inhibitors) have produced robust conditioned place preference. This indicates that serotonin or serotonin-related mechanisms may mediate reward in the absence of dopamine (Hnasko et al. 2007).

It is well known that addictive drugs like morphine affect numerous neurochemical pathways, including those related to the reward system (Koob 1992). Morphine as MOR (µ-opioid) agonist has been shown to increase dopamine transmission via mechanisms of decreasing the release of GABA onto dopamine neurons in the VTA (Johnson and North 1992).

Acute administration of high doses of morphine (over 1.0 mg/kg) inhibited or did not evoke 50-kHz USVs (Nagai et al. 2004; Wright et al. 2012b; Simola et al. 2012; Hamed et al. 2012). Our previous studies showed that when morphine was administered for 14 days (once a day), an increase in the number of USVs episodes, recorded after drug injection, was observed on the 14th day as compared to the 1st and the 7th day (Hamed et al. 2012). Additionally, we have demonstrated that morphine-treated rats emitted much more ultrasonic vocalization in 50-kHz band in response to the context of morphine administration after withdrawal period compared to the same measurement before withdrawal period (Hamed et al. 2012). The neurochemical cause of this behavioral phenomenon still remains unknown. Moreover, several studies have shown that 50-kHz USVs are emitted during anticipation of the natural, pharmacological reward or even rewarding electrical brain stimulation (Burgdorf et al. 2000; Knutson et al. 1999; Opiol et al. 2015; Buck et al. 2014; Hamed et al. 2012). Simultaneously, existence of individual differences in the emission of 50-kHz USVs between rats further complicates the interpretation of behavioral data. Nevertheless, the fact that there are individual differences in the emission of the 50-kHz USVs allows us to explore neurochemical differences in structures related to reward processing, enabling us to explore biological basis of that phenomenon.

Taking into account that morphine pre-treated rats emitted much more context-induced 50-kHz USVs on the challenge day (after 14 day withdrawal period) and the aforementioned literature facts about pharmacological modifications of 50-kHz USVs we conducted non-parametric analysis between concentrations of neurochemical compounds in several brain structures (see “Materials and methods”) with the level of context-induced conditioned 50-kHz USVs. We hypothesized that the 50-kHz USVs emission could have separate neurochemical background in different behavioral paradigms as well as some common neurochemical mechanisms reflected in examined neurotransmitters correlations.

The aims of this study were to: (1) extract the most important neurotransmitters that are bound up with context-induced conditioned 50-kHz USVs related with pharmacological reward; (2) find biological basis of occurring inter-individual differences in context-induced conditioned 50-kHz USVs response; (3) create a model of *all-to-all* neurotransmitters correlations.

## Materials and methods

### Animals

Adult male Sprague–Dawley rats (*n* = 38; 180 ± 20 g) were used in the experiment. The animals were purchased from a licensed breeder (the Polish Academy of Science Medical Research Center, Warsaw, Poland). The animals were housed in standard laboratory conditions under 12 h:12 h light:dark cycles (lights on at 7 a.m.) at a constant temperature (21 ± 2 °C) and 70% humidity. The rats had free access to food and water. The experiments were performed in accordance with the European Communities Council Directive of 24 November 1986 (86/609 EEC). The Local Committee for Animal Care and Use of Warsaw Medical University approved all experimental procedures using animal subjects.

### Experimental procedure

Groups of five animals were housed in acrylic cages (cage size: 54 cm × 34 cm × 21 cm) for 2 weeks. Morphine in dose 10.0 mg/kg (s.c.) was administered repeatedly to the experimental groups. Morphine and saline were administered in testing cages in a group of four animals for each cage. The testing room was significantly different than home cage room, both in the lighting conditions and in the arrangement of spatial cues that were constant throughout the whole experiment. The testing room was situated in a remote part of the laboratory. In Morph-D14 group, 13 injections were administered once a day. In the Morph-D28 group morphine was administered once a day for 14 days (10 mg/kg s.c). Saline solution was administered repeatedly (1.0 ml/kg) to control groups (Saline-D14 and) in the same manner as morphine in morphine treated rats. All animals were kept for 30 min in the testing box after each saline or morphine injection.

**Fig. 2:**
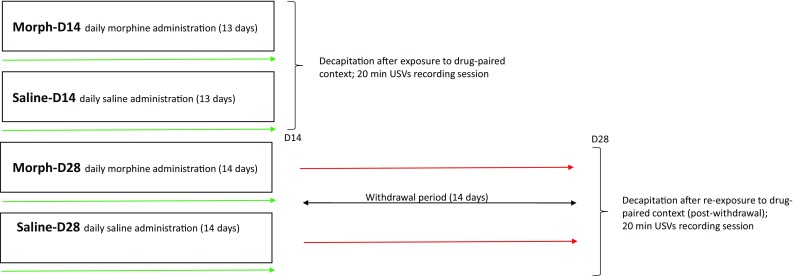
Diagram of the experiment

On day 14, Morph-D14 group and control Saline-D14 group were exposed to the context of drug administration, and the ultrasonic vocalizations were recorded for 20 min. Immediately after 20 min of USVs recording session rats from Morph-D14 and Saline-D14 groups were decapitated. On day 14, 30 min after last injection Morph-D28 and Saline-D28 group were left undisturbed (in their home cages), whilst they were subjected to 2-week withdrawal period. On day 28, the rats were re-exposed to the context of morphine/saline administration and ultrasonic vocalization was recorded for 20 min. Immediately after 20 min of USVs recording session rats from Morph-D28 and Saline-D28 groups were decapitated (Fig. 2). The USVs were recorded in a dark room with a dim red light [30 W bulb 1.5 m above the acrylic cage (size: 54 cm × 34 cm × 21 cm)]. The USVs response to the context was measured separately for each rat. To avoid the influence of scent marks from other animals on the behavior of subsequently tested rats, cages were cleaned with a 70% ethanol solution after each recorded session. Brain tissues were frozen in dry ice-cold isopentane, and stored at − 70 °C for neurochemical analysis.

### Drugs

Morphine hydrochloride (Polfa, Poland) was dissolved in 0.9% isotonic saline. Morphine was administered in dose of 10.0 mg/kg (1.0 ml/kg; s.c.). Saline was used as a control vehicle (1 ml/kg).

### Apparatus and USVs recordings

All subtypes of 50 kHz rat calls were recorded using an UltraSoundGate Condenser Microphone CM16 (Avisoft Bioacoustics, Berlin, Germany) that was positioned 25–30 cm above the floor of the cage. This microphone was sensitive to frequencies of 15–180 kHz with a flat frequency response (± 6 dB) between 25 and 140 kHz. It was connected to an amplifier (custom-made) that had the following parameters: a voltage gain of 16 V/V (12 dB), a frequency response of ± 0.1 dB, a range of 30 Hz to 120 kHz, and an input impedance of 600Ω. The signal was then transferred through a 120 kHz anti-aliasing filter (custom-made). The filtered sounds were sent to a PCI-703-16A data acquisition board (Eagle Technology, USA). This board was a 14-bit 400-kHz analogue input and analogue output board for PCI-based systems. The recorded data were processed using the RAT-REC PRO 5.0 software (custom-made). The signals were processed through a fast Fourier-transformation (1024, Hamming window) and displayed as color spectrograms. Each signal was manually marked with the section label included in the automated parameter measurement. Various parameters were determined automatically, including the number of USV calls, the total calling time (s), the mean call length (s), the frequency bandwidth (kHz), the number of gaps, the mean gap length (s), and the mean peak frequency (kHz). Only the number of calls is included as a main parameter in the results presented here. Taking into account that dopaminergic system plays role in the processing of both appetitive and aversive states (Bromberg-Martin et al. 2010; Zweifel et al. 2011; Lammel et al. 2011a, b), we have analyzed FFT spectrograms in whole recorded frequency spectrum (10–130 kHz) to evaluate occurrence not only “50-kHz appetitive”, but also “22-kHz aversive” calls. Detailed analysis of the FFT spectrograms showed the absence of 22-kHz (alarm calls) in presented model.

### HPLC analysis of monoamines

Frozen brains were cut into slices (− 20 °C) in a cryostat. The following structures were dissected: prefrontal cortex (3.7–3.2 mm anterior to the bregma); nucleus accumbens (NAcc) and caudate putamen (CPu) (1.70–1.0 mm anterior to the bregma); hippocampus and amygdala (− 2.80 to − 3.60 mm posterior to the bregma); and ventral tegmental area (− 5.80 to − 6.30 mm posterior to the  bregma) based on the rat brain atlas of Paxinos and Watson (1998). Tissue samples were weighed and homogenized for 30 s in 15 volumes of ice-cold 0.2 M perchloric acid, which contained dihydroxybenzylamine as an internal standard. The homogenates were then centrifuged at 26,900*g* for 8 min at 4 °C. The supernatants were then filtered through 0.45-μm pore filters and stored at − 70 °C until analyzed for noradrenaline (NA), 3-methoxy-4-hydroxyphenylglycol (MHPG), dopamine (DA), 3,4-dihydroxyphenylacetic acid (DOPAC), homovanillic acid (HVA), 3-methoxytyramine (3-MT), serotonin (5-HT) and 5-hydroxyindoleacetic acid (5-HIAA) using HPLC, as described by Kaneda et al. (1986), with minor modifications (Szyndler et al. 2010). The concentrations of NA, MHPG, DA, DOPAC, 3-MT, HVA, 5-HT and 5-HIAA were calculated as ng/g of brain tissue. Additionally, to approximate their turnover, the following concentration ratios were computed: 3-MT to DA, DOPAC to DA, HVA to DA, 5-HIAA to 5-HT and MHPG to NA.

### HPLC analysis of amino acids

The brain levels of the amino acids were determined using HPLC method, coupled to an electrochemical detection, as described previously (Szyndler et al. 2008). Briefly, the compounds were eluted isocratically with the mobile phase delivered at 0.7 ml/min, using a Shimadzu Class LC-10ADvp pump. An electrochemical detector with a flow-through cell (Intro-Antec Leyden) linked to the Shimadzu Class VP Integrator SCL-10 Avp was used. A high-density glass carbon-working electrode (Antec) was used at + 0.85 V. A Rheodyne injection valve with 20-µl sample loops was used to manually inject the samples. The preparation of the mobile phase and the derivatizing agents was based on the slightly modified method of Rowley et al. (1995; Szyndler et al. 2008). The concentrations of GABA, alanine, taurine, glutamine and glutamate were calculated in µmol/g of tissue. Similarly as in case of monoamines, ratios of the glutamate concentration to both glutamine and GABA were also computed and investigated.

### Statistics

All levels of compounds across brain structures and the aforementioned level ratios formed a set of 130 descriptors of the brain state. One should note, however, that the introduction of ratios has created certain artificial correlations, disallowing descriptor independence assumptions.

According to a Shapiro–Wilk test, distributions of most (53%) of the predictors analyzed in this study are not normally distributed, hence non-parametric statistical approaches were predominantly used.

Relations between numerical variables were assessed using the Spearman correlation, as it is not only robust to non-normality, but can be effectively assessed in terms of significance assuming independence as a null hypothesis, even exactly for small sample sizes.

On the other hand, relations between numerical and binary variables were assessed using the Mann–Whitney–Wilcoxon test.

Still, these tests do not consider possible multivariate associations; to this end, the Boruta machine learning method was employed (Kursa and Rudnicki 2010).

It works by iterative fitting of the Random Forest (Breiman 2001) model to the data, and extracting variables that are significantly more useful for that purpose than shadows, design injected into the dataset. When not specified otherwise, Holm–Bonferroni method was used to correct for multiple comparisons, and the significance level was set at *p* = 0.05. All computations were performed using R 3.4.1 (R Core Team 2017) with the Boruta 5.2.0 (Kursa and Rudnicki 2010), pspearman 0.3–0 (Savicky 2014) and ranger 0.8.0 packages (Wright and Ziegler 2017).

## Results

### The effects of morphine administration and withdrawal period on context-induced conditioned 50-kHz USVs

**Fig. 3:**
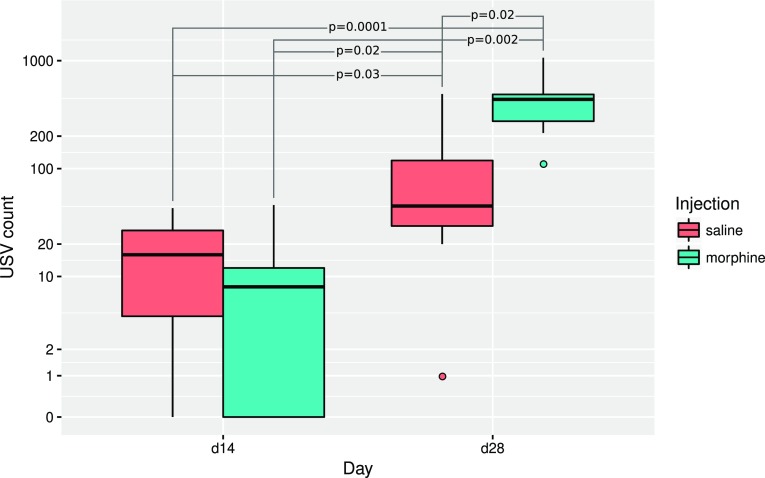
Observed “50-kHz” USV episode counts (number of episodes), for the 14th (d14) and 28th (d28) day of the experiment, and for saline- and morphine-injected rats. USV count is shown using an inverse hyperbolic sine (IHS) scale. Bars mark significant differences according to Mann–Whitney–Wilcoxon test

**Fig. 4:**
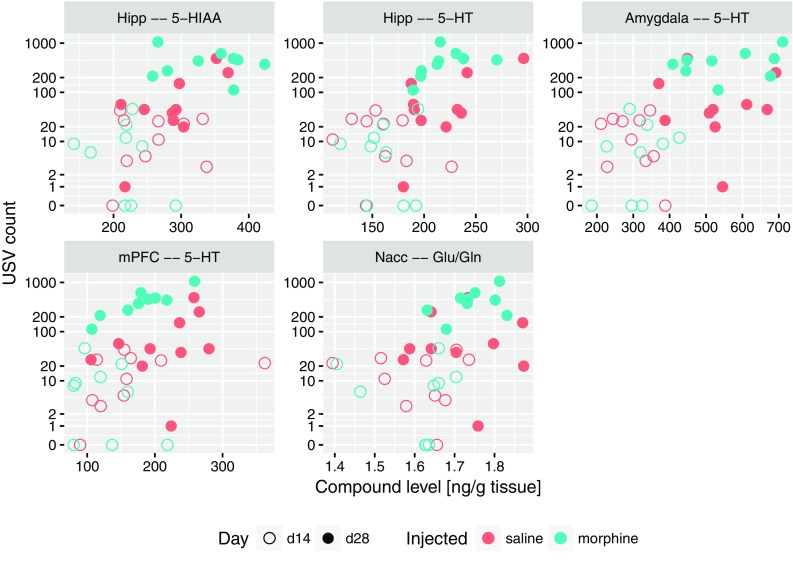
Relation between USV count (number of 50-kHz USVs episodes) and levels of compounds or level ratios which were significantly correlated with it, shown as scatterplots. USV count is shown using IHS scale

The effects of morphine and withdrawal period on context-induced conditioned 50-kHz USVs count has been analyzed with a series of 6 Mann–Whitney–Wilcoxon tests, and corrected for multiple comparisons. The only non-significant difference between USV counts (number of 50-kHz USVs episodes) in experimental groups was observed between saline and morphine groups on day 14. Between day 14 and day 28, the USV count has increased both in saline (*p* = 0.03) and morphine (*p* = 0.002) groups, while on day 28 the morphine-treated rats have vocalized more frequently than saline-treated (*p* = 0.02) (Figs. 3, 4).

### The correlations between USV counts and neurotransmitter levels

Next, the links between USV count and levels or level ratios of the analyzed compounds in across brain structures were assessed. To this end, Spearman correlation test was used, also allowing for extracting the sign of correlation. Additionally, the Boruta machine learning method was used to investigate potential multivariate interactions. Such analysis was performed for all rats, as well as in context of morphine- and saline-treated group.

The strongest, significant correlations with USV count were: with 5-HT level in amygdala, hippocampus (for all rats) and medial prefrontal cortex (for morphine group), as well as with 5-HIAA in hippocampus (for all rats). Furthermore, we found USV count to be significantly correlated with the Glu/Gln ratio in nucleus accumbens for morphine group. The raw data behind this interactions are shown on Fig. 4. All these results were also confirmed by the Boruta analysis, along with a number of lesser interaction, collected in Table 1.

**Table 1:**
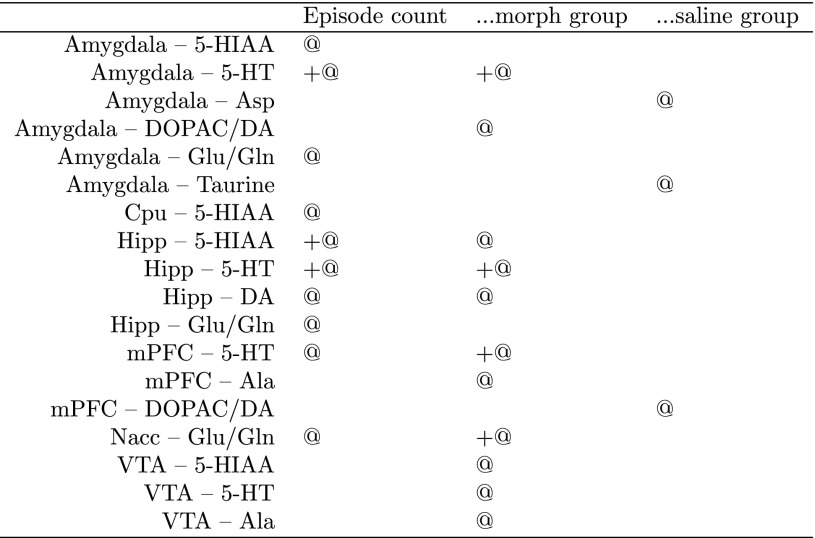
The selection of brain compounds which level or level ratio is significantly linked with the USV count

“+” denotes positive correlation, “@” association identified with the Boruta method

### The effect of the re-exposure to the morphine-paired context on neurotransmitters levels

Similar analysis was performed to analyze which compounds can be linked with context-induced conditioned response. This is a classification problem, hence Mann–Whitney–Wilcoxon test was used instead of Spearman correlation. The Boruta method can handle both classification and regression problems, so the auxiliary analysis involving this approach was performed exactly in the same way as in the previous case. Again, contexts of all rats, morphine and saline groups were investigated separately. Especially, we found positive associations between context conditioning and serotonin levels in amygdala, hippocampus and mPFC, as well as with Glu/Gln ratio in nucleus accumbens, both for all rats and only morphine group. The other interesting effects are: increase of Glu/GABA ratio in VTA and in amygdala for all rats, increased glutamate in amygdala for all rats and only for morphine group, decrease of GABA in VTA and mPFC for all rats and only for morphine group. The said analysis yield numerous significant results, which are summarized in Table 2.

### The effects within neurotransmitter levels

**Table 2:**
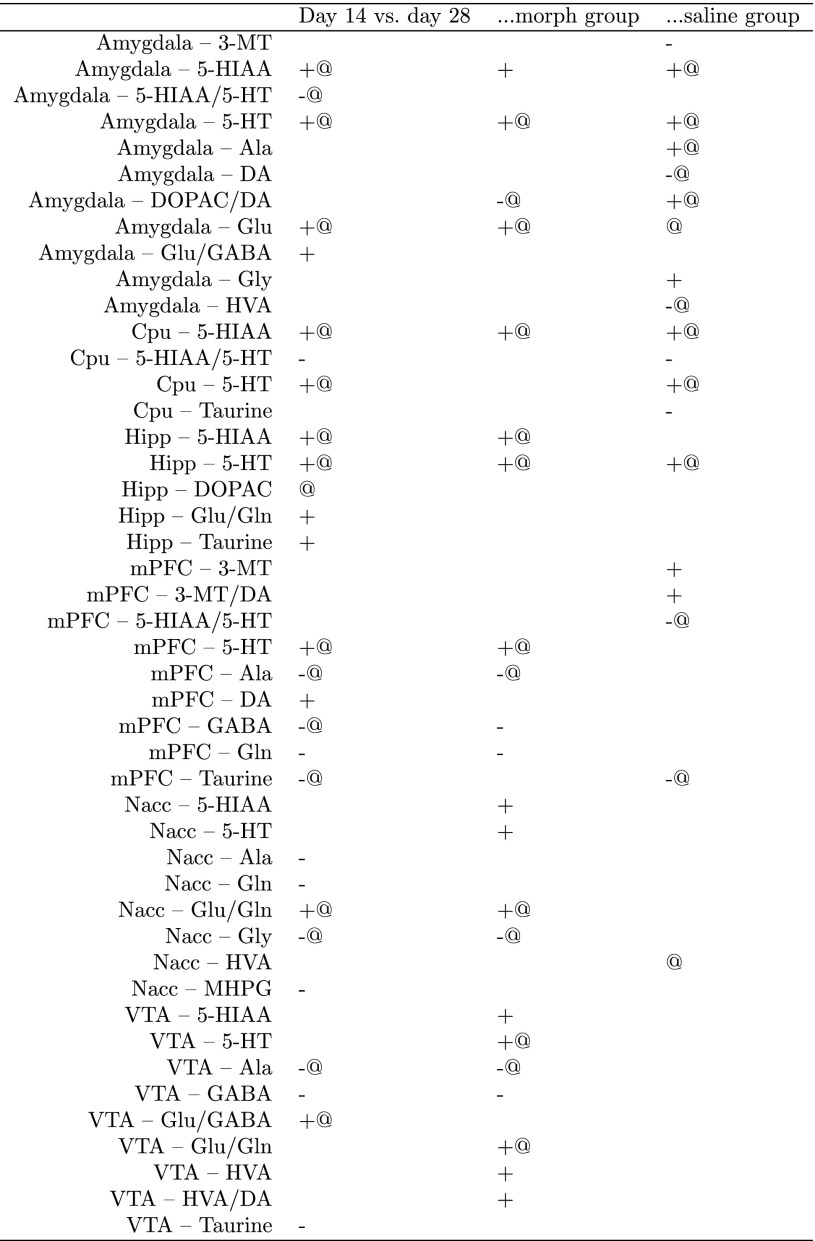
The selection of brain compounds which level or level ratio is significantly linked with post-withdrawal conditioned response to the context

“+” denotes positive correlation, “−” negative, while “@” association identified with the Boruta method

**Fig. 5:**
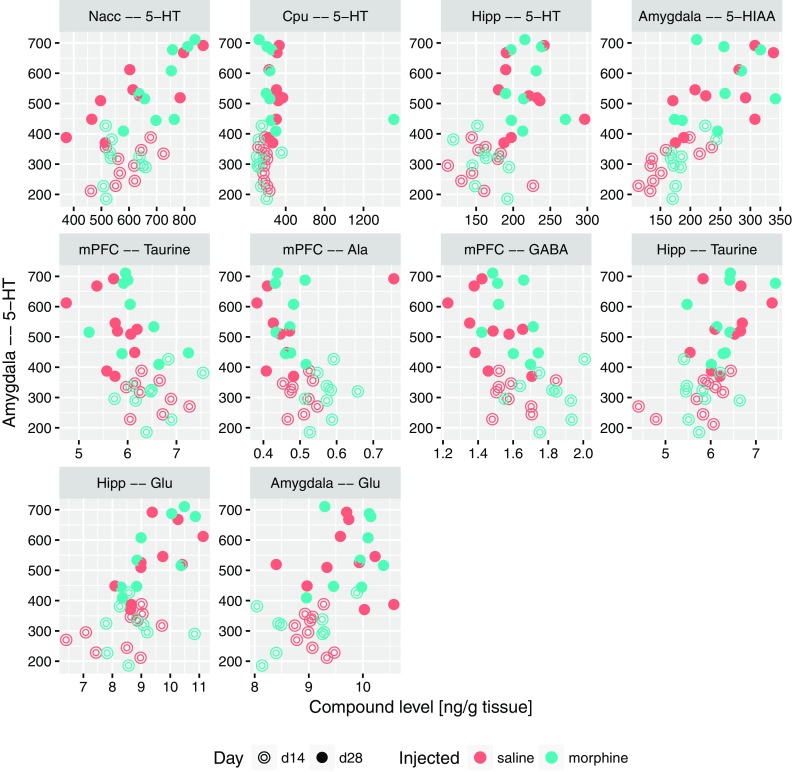
Relation between the level of 5-HT in amygdala and levels of compounds which were significantly correlated with it, shown as scatterplots

**Fig. 6:**
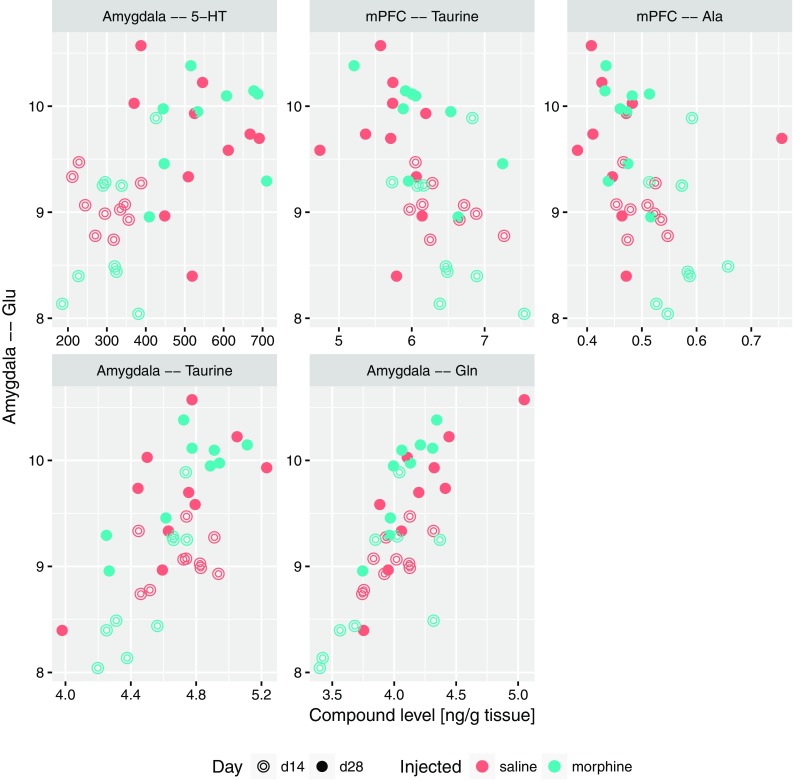
Relation between the level of glutamate in amygdala and levels of compounds which were significantly correlated with it, shown as scatterplots

**Fig. 7:**
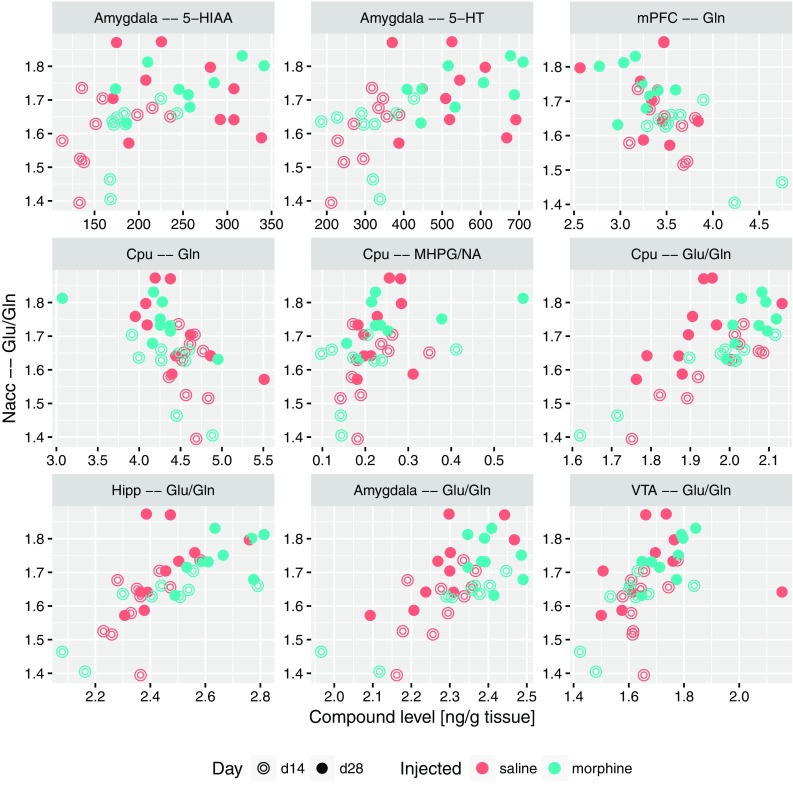
Relation between the ratio of glutamate to glutamine level in nucleus accumbens and levels of compounds or level ratios which were significantly correlated with it, shown as scatterplots

**Fig. 8:**
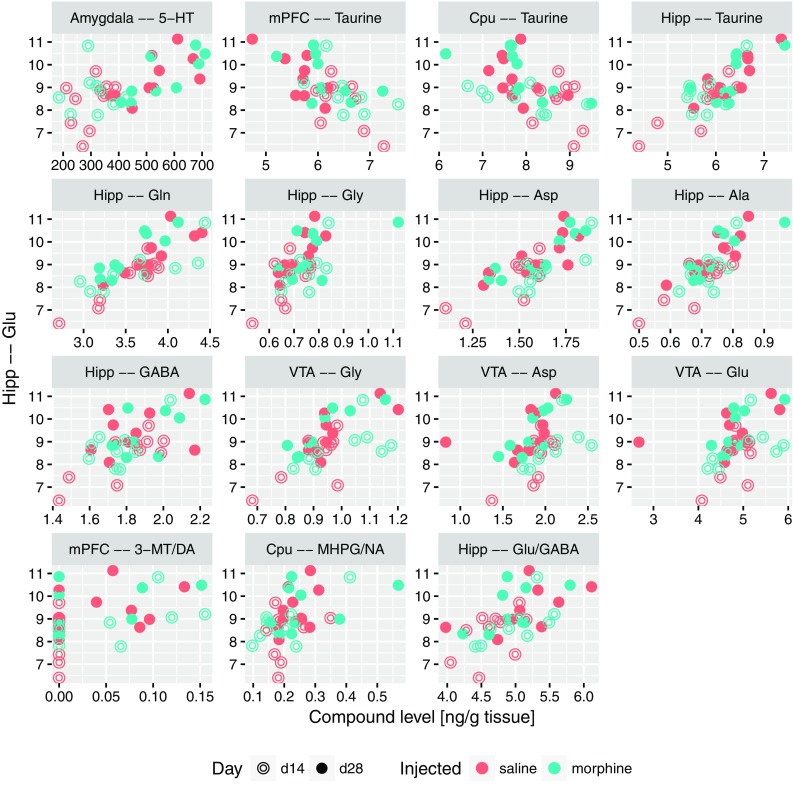
Relation between the level of glutamate in hippocampus and levels of compounds which were significantly correlated with it, shown as scatterplots

Based on the results highlighted above and theoretical considerations, four substantial descriptors were selected: 5-HT and glutamate levels in amygdala, the ratio of glutamate and glutamine levels in nucleus accumbens, finally the levels of glutamate in hippocampus. For these, sets of other descriptors that significantly correlated with each of them were identified. To this end, Spearman correlation-based methodology identical to previously used for associations with the USV count was applied. All significant correlations with the serotonin level in amygdala are presented in Fig. 5; they include positive correlations with 5-HT levels in other structures (Nacc, Cpu and hippocampus), serotonin metabolite 5-HIAA in amygdala, as well as with the levels of glutamate in both amygdala and hippocampus. On the other hand, negative correlations were observed with the levels of taurine, alanine and GABA in medial prefrontal cortex. Figure 6 contains all the significant correlations with the level of glutamate in amygdala; similarly, it was correlated positively with levels of 5-HT and glutamine, and negatively with levels of alanine and taurine in mPFC. A positive correlation with the level of taurine in amygdala was also detected. The correlations of Glu/Gln ratio in nucleus accumbens are collected in Fig. 7. This set contains positive correlations with the same ratio in other structures: amygdala, Cpu, hippocampus and VTA, as well as negative with glutamine in mPFC and Cpu; also a positive correlation with serotonin and 5-HIAA in amygdala was identified. An interesting finding is a correlation between Glu/Gln in Nacc and MHPG/NA ratio in Cpu. Finally, all significant correlations of Glu levels in hippocampus are collected in Fig. 8; they include positive correlations with: MHPG/NA ratio in Cpu; GABA, glutamine, taurine level and Glu/GABA ratio in hippocampus; glutamate level in VTA, serotonin concentration in amygdala.

### Intra-neurotransmitter level correlation structure

**Fig. 9:**
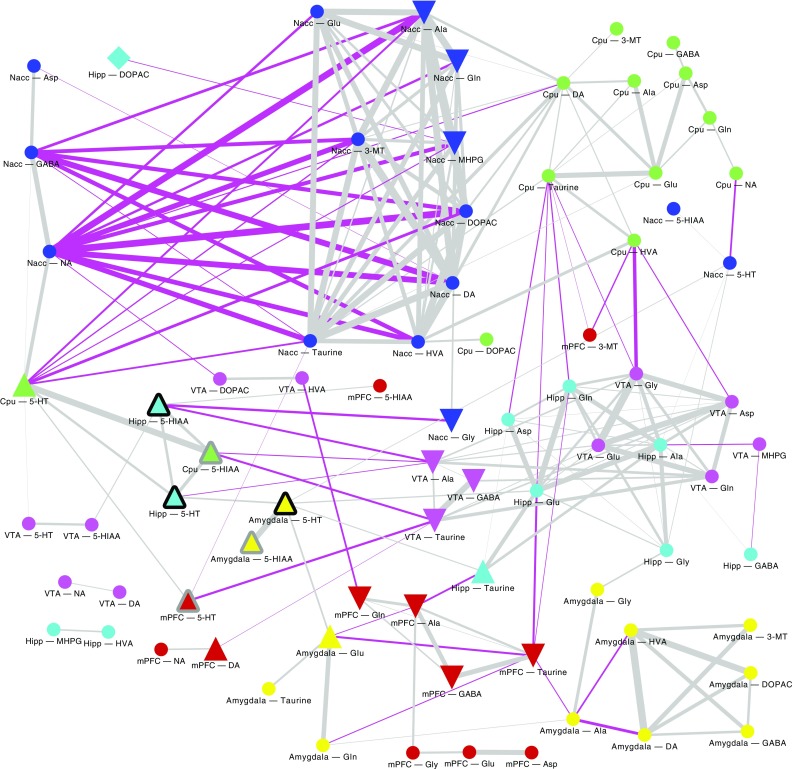
Graph of significant correlations between compound levels. Node shape denotes significant relation with post-withdrawal conditioned response to the context, respectively: triangle up—level elevated in post-withdrawal conditioned response to the context, triangle down—lowered, square—interaction identified exclusively by machine learning. Node outline denotes significant relation with the USV episode count, respectively: black outline—positive correlation, grey outline—interaction identified exclusively by machine learning. Edge with corresponds to the correlation strength, while its colour to the correlation sign: grey denotes positive, while fuchsia negative. Node colours denote brain structure

Finally, a global, all with all correlation scan was performed. To avoid artificial correlations, the ratios were discarded for this analysis. It was also performed using Spearman correlation test, although the Benjami Hochberg’s multiple comparison correction (FDR) (Benjamini and Hochberg 1995) was used. The results of conducted analysis were compiled into a graph, nodes of which correspond to compound levels across structures, while edges to correlations significant at *p* = 0.03 level. The graph was manually laid out to expose relevant coherent structures, and presented in Fig. 9.

## Discussion

The data collected here confirms our previous behavioral results that re-exposure to the context of morphine administration after the withdrawal period (day 28) increases the level of 50-kHz USVs episodes, compared to day 14 (Fig. 3) (Hamed et al. 2012). Context-induced 50-kHz USVs emission is variable among rats, what indicates individual differences in context conditioned response. This kind of gradation allows us to analyze individual differences in brain neurotransmission and understand the basis of occurring variations in response to the context.

Interestingly, in control saline group an increase of 50-kHz USVs was also observed on day 28 compared to day 14. One of the reasons might be that both morphine and saline were administered in testing cages in a group of four animals for each cage (see “Materials and methods”). This explanation could suggest that the context conditioned response was enhanced by the natural social contact associated with a different, uncommon environment. Considering rewarding effects of morphine reflected by 50-kHz USVs emission reported in our previous studies (Hamed et al. 2012), we can be sure that emitted sounds reflect context-induced conditioned response related to morphine rewarding effects. This kind of behavioral 50-kHz USVs response was reported in studies with natural, pharmacological reward or even with rewarding electrical brain stimulation (Knutson et al. 1999; Burgdorf et al. 2000; Opiol et al. 2015; Buck et al. 2014). The level of emitted 50-kHz USVs was over ten times higher in morphine-treated group than in saline group and only three of saline control animals produced more than hundred USVs episodes (Figs. 3, 4). The starting point of our correlations analysis was the level of context conditioned response reflected by 50-kHz USVs emission. All further consecutive analysis were conducted in the same way in the morphine and saline-treated animals (Figs. 4, 5, 6, 7, 8, 9; Tables 1, 2).

Nevertheless, the fact that emission of 50-kHz USVs is a context-induced conditioned response, it is interesting why re-exposure to the drug-paired context did not evoke such sounds on day 14 in the same manner as on day 28 (after 14 day-withdrawal period; Figs. 3, 4). This time-dependent differentiation effect of observed behavior may be explained by the phenomenon defined as “incubation of drug craving”. It was demonstrated in humans that craving may be triggered by drug-associated cues and may increase progressively during the early days of withdrawal (Gawin and Kleber 1986; Abrams et al. 1988; Drummond et al. 1990; George et al. 2001; Drummond 2000; Li et al. 2012). In rodents, incubation of craving has been demonstrated both in drug-treated animals (Grimm et al. 2001; Shalev et al. 2001; Shepard et al. 2004; Abdolahi et al. 2010) as well as in non-drug reward experiments (Grimm et al. 2002). Incubation of craving involves neuroadaptations in reward and motivation-related structures (Conrad et al. 2008; Sesack and Grace 2010; Wolf and Tseng 2012; Lee et al. 2013; Purgianto et al. 2013; Ma et al. 2014; Pascoli et al. 2014; Li et al. 2015; Wolf 2016). It is well known that nucleus accumbens is key structure of the limbic system that is highly related to reward processing (Berridge 2007; Ikemoto 2007) and 50-kHz USVs emission (Thompson et al. 2006; Brudzynski 2013a, b; Hamed et al. 2016).

The present study shows that 50-kHz USVs emission is associated with increased serotonin concentrations in amygdala, hippocampus and mPFC and also with elevated Glu/Gln ratio in nucleus accumbens (Fig. 4; Table 1). Moreover, concentration of serotonin in amygdala is directly proportional with the level of serotonin in nucleus accumbens and in hippocampus as well as with concentration of glutamate in amygdala and hippocampus (Fig. 5).

It was found that central amygdala (CeA) plays a critical role in incubation of drug and non-drug reward craving (Uejima et al. 2007; Li et al. 2008, 2015). Inhibition of CeA neuronal activity reduced incubation of nicotine, cocaine, methamphetamine and sucrose craving (Funk et al. 2016; Lu et al. 2005, 2007; Uejima et al. 2007; Li et al. 2015). Human fMRI studies report that amygdala is the key structure in the generation of cue-elicited opioid craving (Langleben et al. 2008, 2014; Mei et al. 2010; Li et al. 2012; Murphy et al. 2017). It was found that GABA-ergic inhibition of neuronal activity leads to reduction of behavioral expression of craving (Li et al. 2015). It was also demonstrated that ethanol withdrawal reduced GABA levels in medial prefrontal cortex and enhanced glutamate and glutamine levels in NAcc (Hinton et al. 2012). In our study, we found that GABA as well as alanine and taurine concentrations in mPFC are inversely proportional to the concentration of serotonin in the amygdala (Fig. 5) and the concentration of GABA in mPFC was decreased after withdrawal period. These data indicate that reduction of GABA-ergic inhibition in mPFC may be one of the initiating factors of drug-seeking behavior and expression of craving reflected by 50-kHz USVs. We have also demonstrated for the first time that Glu/GABA ratio in VTA and amygdala was elevated after re-exposure to the drug-paired context on the day 28 compared to day 14 (Table 2).

It was demonstrated in optogenetic studies that silent synapse-based reorganization of the amygdala-to-accumbens projections plays key role in stability of cocaine craving and relapse after withdrawal period (Lee et al. 2013). Furthermore, it was found that mechanisms related to the action of MMP-9 (an important controller of the synaptic plasticity of excitatory synapses) in central amygdala are crucial in generating a motivation for reward seeking (Stefaniuk et al. 2017). An elevated glutamate concentration in amygdala which correlates with increased serotonin in this structure (Fig. 6), parallels an experiment in which the reduction of serotonergic neurotransmission in amygdala promoted hyperexcitability of this structure by enhancing glutamatergic neurotransmission, in consequence increasing fear related behaviors (Tran et al. 2013). It might further indicate that increased concentration of serotonin in this structure presented in our study, prevents or switches over the arousal from fear expression to appetitive arousal related to drug-seeking behavior expressed by 50-kHz emission. Further investigations of mentioned neurochemicals co-existence in the amygdala have to be performed to address this hypothesis. Moreover, ex vivo electrophysiological recordings with optogenetic methods and pharmacological analysis revealed the existence of the 5-HT and glutamate co-transmission in basal amygdala neurons (Sengupta et al. 2017).

The very nature of the conditioned place preference is attributed to activation of the reward system and its association with information on the surrounding space during training sessions. This information in the hippocampus is represented by a subset of spatially tuned neurons called *place cells* (Hollup et al. 2001). The hippocampus and its inputs have been implicated in memory formation, including reward-related memory (Scoville and Milner 1957; Lisman and Grace 2005; Hernández-Rabaza et al. 2008; Bunzeck et al. 2011). Furthermore, it was demonstrated that a projection from area CA3 of dorsal hippocampus to ventral tegmental area (VTA) mediated relations between context and reward (Luo et al. 2011). Ntamati and Luscher identified and characterized a projection from VTA that releases both glutamate and GABA near the granule cells of hippocampal DG (Ntamati and Lüscher 2016).

We found that the level of serotonin in amygdala is directly proportional to the concentration of glutamate in amygdala and hippocampus as well as to the level of serotonin in nucleus accumbens and in the hippocampus (Fig. 5). Additionally, *all-to-all* analysis indicate that concentration of glutamate in hippocampus is directly proportional to glutamate level in VTA and GABA concentration in the hippocampus (Figs. 8, 9).

There are potent structural interconnectivities and functional relationships between nucleus accumbens (Nacc), amygdala and hippocampus. Nucleus accumbens integrates cortical and limbic glutamatergic inputs arising from basolateral amygdala (BLA), hippocampal ventral subiculum (vSub) and prefrontal cortex (PFC) (Phillipson and Griffiths 1985; Groenewegen et al. 1987; McDonald 1991; Shinonaga et al. 1994; Johnson et al. 1994; O’Donnell and Grace 1995; Petrovich et al. 1996; Friedman et al. 2002; French and Totterdell 2003; Sesack and Grace 2010; Gill and Grace 2011; Britt et al. 2012).

It is well known that the amygdala is a structure involved in expression of the emotion and in learned emotional behaviors (LeDoux 2000). Previous studies have established that the BLA is involved in affective response and the vSub in context dependency (Gill and Grace 2011). Optogenetic studies revealed that selective activation of BLA, but not mPFC, glutamatergic inputs to the NAcc promotes motivated behavioral responding (Stuber et al. 2011). On the other hand, photostimulation of each of the different afferent pathways (vHipp, mPFC, BLA axons) to the NAcc reinforced instrumental behavior (Britt et al. 2012). Britt and colleagues suggested that the specific pathway releasing glutamate is not as important as the amount of glutamate that is released (Britt et al. 2012). Anatomical and functional interconnectivity of the amygdala and hippocampus has been demonstrated in a number of studies (Mello et al. 1992a, b; Maren and Fanselow 1995; Ikegaya et al. 1994, 1996a, b; Akirav and Richter-Levin 1999; Nakao et al. 2004).

We did not detect correlative association of glutamate level between hippocampus and amygdala, but we found strong relation between glutamate concentration in hippocampus and the serotonin level in amygdala (Figs. 8, 9). Considering the fact that the hippocampus is strictly involved in spatial memory formation, we can suppose that, after or during activation of hippocampus-VTA loop the hippocampus sends glutamatergic signal to the amygdala to amplify the serotonergic signaling. Then, 5-HT augmentation may initiate or support the glutamatergic signaling in amygdala. These processes might have a strong impact on the 50-kHz USVs expression of emotions reflected in neurochemical changes in Glu/Gln ratio in Nacc. Understanding the sequence of activation of the neurochemical signaling between interacting structures requires further research using electrophysiological, optogenetic or voltammetric methodology.

Obtained neurochemical data confirms the contribution of amygdala, nucleus accumbens and hippocampus to spatial memory formation and memory processing bound up with enhanced emotional states (arousal) triggered by an appetitive conditioned context (Hall et al. 2001; Ito et al. 2006, 2008). Taking into account the strong serotoninergic response simultaneously in amygdala, nucleus accumbens, mPFC and hippocampus in processing context conditioned response, based on literature reports, it is reasonable to assume that the structure responsible for this increased levels of 5-HT was dorsal raphe. This structure represents one of the most sensitive reward sites in the brain (Yi Li et al. 2016; Luo et al. 2015, 2016; Matthews et al. 2016; Qi et al. 2014). It has been shown that the nucleus accumbens receives serotonin and non-serotonin inputs from dorsal raphe nucleus (Van Bockstaele and Pickel 1993; Brown and Molliver 2000). Unfortunately, because we did not expect such strong serotonin response in case of 50-kHz USVs emission, we did not analyze concentrations of the neurochemical compounds in dorsal raphe.

Rewards such as food, sucrose, social interaction or sex rapidly activate serotonin neurons in dorsal raphe (Yi Li et al. 2016). It was also demonstrated that dorsal raphe neurons encode reward via serotonin and glutamate (Liu et al. 2014). It is well known that SSRIs affecting the serotonergic system have been used extensively in the treatment of psychiatric disorders over past 20 years. In the studies using microdialysis methods, acute treatment with citalopram increased extracellular serotonin concentration in the central amygdala to 175% (Bosker et al. 2001) of basal level and in the ventral hippocampus citalopram increased serotonin to 325% of the basal level (Cremers et al. 2000). It is also interesting that studies on dopamine-deficient mice have demonstrated that dopamine is not required for morphine-induced reward as measured by conditioned place preference (Hnasko et al. 2005), which indicates that dopaminergic mesolimbic system is not crucial for reward processing and acquisition. Hnasko et al. (2007) demonstrated that in mice with dopamine deficiency the fluoxetine at dose of 5.0 mg/kg produced robust conditioned place preference. This indicate that serotonin may mediate reward in the absence of dopamine (Hnasko et al. 2007).

It is well known that glutamate is the primary excitatory neurotransmitter in the nervous system (Sladeczek et al. 1985). Biochemical studies demonstrate that astrocytic glutamine plays a crucial role in sustaining excitatory neurotransmission (Hertz 1979; Tani et al. 2014). Given the presence of the physiological glutamate–glutamine cycle (Hertz 1979; Scofield and Kalivas 2014; Tani et al. 2014) we have analyzed the correlation of behavioral and biochemical data with Glu/Gln ratio in all examined structures. Analysis of another neurotransmitter/metabolite ratios were also done (see “Statistics”).

We have shown for the first time that the number of USVs episodes strongly correlates with Glu/Gln ratio in nucleus accumbens (Fig. 4) and the increased Glu/Gln ratio in nucleus accumbens has strong associations with Glu/Gln ratio simultaneously in VTA, amygdala, CPu and hippocampus (Fig. 7). This parameter also positively correlates with concentrations of serotonin and its metabolite 5-HIAA in amygdala as well as with MHPG/NA ratio in CPu (Fig. 7). Increased levels of Glu/Gln ratio in amygdala presented in our study is interesting in light of data from electrochemical studies, which indicate that glutamate concentration in basolateral amygdala is transiently elevated by reward-predictive stimuli (Malvaez et al. 2015). Moreover, previous studies showed that blockade of the glia selective glutamate reuptake in the amygdala and in VTA induced depressive-like effects, including anhedonic symptoms manifested in reduced social interactions and reduced sensitivity to reward (Herberg and Rose 1990; Lee et al. 2007; Bechtholt-Gompf et al. 2010; John et al. 2012). It was also demonstrated that dentate gyrus of the hippocampus was the main structure responsible for this effect and impaired spatial memory was related to observed anhedonic symptoms (Bechtholt-Gompf et al. 2010). Furthermore, it was reported that decreased levels of astrocytic glutamate transporter occurred in animal models of depression (Zink et al. 2010). It is worth mentioning that the most commonly used anti-depressive drug fluoxetine (selective inhibitor of the serotoninergic transporter) induced astrocytic glutamate transporter expression in hippocampus, amygdala and retrosplenial granular cortex (Zink et al. 2011).

Thus, the relationship between the emission of 50-kHz USVs and the neurochemical changes that occur after re-exposure to morphine-paired context, indicates strong serotoninergic response in amygdala, hippocampus and mPFC enhanced with increased glutamatergic activity in nucleus accumbens. Most of the studies considering ‘incubation craving’ were carried out with more than 2 weeks of withdrawal period. Nevertheless, the time-dependent differentiation of behavioral response presented in our study indicate that context-induced 50-kHz USVs emission might be a new tool for reflecting individual differences in incubation of craving. Presented analysis indicates a strong correlation between serotonergic and glutamatergic systems in context-induced conditioned response. The strength of this co-transmission correlates with the number of 50-kHz USVs emitted in response to the reward context.

### Limitation of the study

The limitation of the procedure is the lack of control in another environment that was not paired with morphine conditioning. This kind of neutral environment control would exclude chance that 50-kHz USVs might be reflection of incubation of craving triggered without drug-associated spatial cues and the 50-kHz USVs emission might be permanent after 14 days of withdrawal period. Nevertheless, context-induced conditioning USVs was not observed in the first seconds of testing session and these sounds were intensifying with the time spent in the testing cage, probably after acquisition spatial cues.

Another limitation of our study is that the analysis of neurotransmitters concentration was performed post mortem, so there was a time lag between measurement of USV and measurement of neurochemical changes in the brain. However, animals were decapitated immediately after testing session and brain tissue of each animal was isolated strictly after that. It is well known that the advantage of the in vivo technique (voltammetry or microdialysis) is that it enables tracking of changes in the extracellular concentrations of neurotransmitters over time; however, monitoring is generally limited to changes in a single structure. The ex vivo method enables the analysis of not only the extracellular concentration but also the total concentration of neurotransmitters and their metabolites in several brain structures. According to our knowledge, there is no technological possibility to analyze concentration of many neurotransmitters and their metabolites in several structures in vivo in freely moving rats.
